# Leukocyte telomere length and amyotrophic lateral sclerosis: a Mendelian randomization study

**DOI:** 10.1186/s13023-021-02135-2

**Published:** 2021-12-14

**Authors:** Kailin Xia, Linjing Zhang, Gan Zhang, Yajun Wang, Tao Huang, Dongsheng Fan

**Affiliations:** 1 Department of Neurology, Peking University Third Hospital, Beijing, China; 2 Beijing Municipal Key Laboratory of Biomarker and Translational Research in Neurodegenerative Diseases, Beijing, China; 3 Department of Epidemiology and Biostatistics, School of Public Health, Peking University, Beijing, China; 4 Key Laboratory of Molecular Cardiovascular Sciences (Peking University), Ministry of Education, Beijing, China; 5 Key Laboratory for Neuroscience, National Health Commission/Ministry of Education, Peking University, Beijing, China

**Keywords:** Amyotrophic lateral sclerosis, Leukocyte telomere length, Mendelian randomization, Risk factors

## Abstract

**Background:**

Observational studies have suggested that telomere length is associated with amyotrophic lateral sclerosis (ALS). However, whether this association is causal remains unclear. In this study, we aimed to explore the causal relationship between leukocyte telomere length (LTL) and ALS by a two-sample Mendelian randomization (MR) approach. Single-nucleotide polymorphisms (SNPs) for LTL were identified through high-quality genome-wide association studies (GWASs). The ALS GWAS summary data (20,806 cases; 59,804 controls) with largest sample size to date was obtained. We adopted the inverse variance weighted (IVW) method to examine the effect of LTL on ALS and used the weighted median method, simple median method, MR Egger method and MR-PRESSO method to perform sensitivity analyses.

**Results:**

We found that genetically determined increased LTL was inversely associated with the risk of ALS (odds ratio (OR) = 0.846, 95% confidence interval (CI): 0.744–0.962, *P* = 0.011), which was mainly driven by rs940209 in the OBFC1 gene, suggesting a potential effect of OBFC1 on ALS. The results were further confirmed by sensitivity analysis with the MR Egger method (OR = 0.647, 95% CI = 0.447–0.936, *P* = 0.050). Analyses by the weighted median method (OR = 0.893, P = 0.201) and simple median method (OR = 0.935, *P* = 0.535) also showed a similar trend. The MR Egger analysis did not suggest directional pleiotropy, with an intercept of 0.025 (*P* = 0.168). Neither the influence of instrumental outliers nor heterogeneity was found.

**Conclusions:**

Our results suggest that genetically predicted increased LTL has a causal relationship with a lower risk of ALS. Protecting against telomere loss may be of great importance in the prevention and treatment of ALS.

**Supplementary Information:**

The online version contains supplementary material available at 10.1186/s13023-021-02135-2.

## Background

Amyotrophic lateral sclerosis (ALS) is a late-onset deadly neurodegenerative disease that is considered an accelerated ageing disease [1]. Previously, a worldwide study showed that the prevalence of ALS increases with ageing of populations, evoking a global socioeconomic burden [2]. Despite the significant effort in exploring the mechanisms underlying ALS, the causes of the disease and corresponding promising therapeutic strategies remain elusive. Identifying risk factors for ALS could help facilitate the development of novel intervention targets in ALS prevention.

Telomeres are regions of repetitive nucleotide sequences at the end of chromosomes in eukaryotes, playing a pivotal role in maintaining chromosomal stability. Leukocyte telomere length (LTL) is recognized as the “molecular clock” that associated with cellular and organisms’ senescence [3]. Recent studies reported a shortened LTL is in causal relationships with developing neurodegenerative diseases, a decline in cognitive function, and other age-related physiological degenerations [4–6]. However, compelling studies have provided controversial evidence for the effect of LTL on ALS [7–9]. A case–control study including 1,241 European patients with ALS revealed that telomeres were 9% longer in patients than in controls [7]. De Felice et al. reported that the telomere length of 50 patients with sporadic ALS was significantly reduced by 15% compared with that of 50 healthy subjects [8]. Furthermore, shorter telomeres were associated with earlier ALS onset in animal models [9]. Regarding the inconsistencies of the current studies, differences in study subjects may be one of the reasons. Between the two epidemiological studies, differences in sample sizes and methodological limitations of observational studies may be two reasons for the opposite conclusions, including interference from confounders, reverse causality, and selection bias. In addition, the different methods for measuring telomere length may also influence the conclusions. Randomized control trial (RCT) is a promising approach to alleviate the concerns of residual confounding and reverse causation. However, RCTs determining the relationship between LTL and ALS risk have not been previously implemented, mainly due to ethical issues. A deeper investigation of the role of LTL in ALS will reveal a further understanding of the pathological mechanism of the disease and provide corresponding therapeutic targets to improve the survival of ALS patients. We hypothesize that prolonged LTL could causally protect individuals from developing ALS. Similarly, protecting against telomere loss could be a novel way of early ALS prevention.

To decipher whether LTL has a causal effect on the risk of ALS, we conducted a two-sample Mendelian randomization (MR) approach, which examines causation by exploiting strong LTL-related genetic variants as instrumental variables (IVs) [10]. The inverse variance weighted (IVW) method was adopted as the primary approach to evaluate the potential causation, and the weighted median method, simple median method, MR Egger method, and MR-PRESSO method were utilized as sensitivity analyses in our MR framework.

## Methods

### GWAS summary data collection and IVs selection

Using available summary results from genome-wide association studies (GWASs), we performed MR to examine the causal relationship between LTL and ALS. We searched PubMed for GWASs of LTL (up to January 2021) and selected genetic variants significantly associated with LTL as IVs in this MR design. Among these studies, two representative LTL-related GWASs were included in our research. The first GWAS was the study with largest sample size that measured LTL by the Southern blot method and was also the most frequently employed LTL GWAS in MR studies, and the second was the study with the most recent publication date and the largest sample size. IVs identified from the first study were regarded as IV-1, while IVs identified from the second study were regarded as IV-2.

IV-1 was derived from a meta-GWAS based on 6 studies with a total of 9190 European individuals enrolled (aged 18–95) [11]. Telomere length was measured by the Southern blot method for the terminal restriction fragment, which is the current gold standard for LTL measurement [12, 13]. The mean telomere length was 6.83 ± 0.65 kb (mean ± SD) in this study. Age, sex, and smoking were adjusted in this meta-analysis. IVs were chosen based on the method described by Haycock et al., the reliability of which has been verified by many large-scale MR estimates [4, 5, 14]. Briefly, single-nucleotide polymorphisms (SNPs) were associated with LTL at the genome-wide significance level, and the corresponding effects on LTL and standard errors were concatenated by Mangino et al. [11]. Sixteen SNPs within the range of ten loci were included after excluding loci with obvious heterogeneity. These SNPs could explain 9.4% of the genetic variation in LTL [11]. The statistical F value of each SNP ranged from 18 to 28 for each SNP [11]. IVs with F values greater than 10 were considered strong instruments that could avoid bias from weak instruments [15].

IV-2 was obtained from the largest GWAS of LTL, which enrolled a total of 78,592 European participants from the EPIC-InterAct, EPIC-CVD and ENGAGE Consortiums. Mean LTL was measured as a continuous variable by quantitative PCR and expressed as the ratio of the telomere repeat number (T) to a single-copy gene (S) [16]. The ages of participants ranged from 18 to 106. Age and sex were adjusted in this GWAS. Twenty SNPs were reported to be associated with ALS with *P* < 5*10^–8^ after false discovery rate (FDR) correction, which could explain 1–2% of the genetic variation in LTL [16]. The F statistic of each SNP ranged from 27 to 205 [16].

The publicly available European-based GWAS summary statistics for ALS genotyped and imputed more than 10 million SNPs in 20,806 ALS cases and 59,804 controls. All patients had onset of symptoms after 18 years of age and were diagnosed at probable or definite levels according to the El Escorial criteria [17].

Independent SNPs with r^2^ < 0.001 and MAF > 0.05 were selected as IVs. SNPs that could not be found in the ALS GWAS summary data were replaced with proxy SNPs in strong linkage disequilibrium (LD) (r^2^ > 0.9) by searching the SNiPA website (http://snipa.helmholtz-muenchen.de/snipa3/) [18]. If a proxy SNP was not reported, the SNP was excluded from downstream MR analysis. Ultimately, ten independent SNPs in IV-1 and fifteen SNPs in IV-2 were obtained for LTL (displayed in detail in Additional file 1: Table S1). According to IV-1 and IV-2, information on the corresponding SNPs was extracted from ALS summary data, including effect alleles, other alleles, effects, standard errors, and P values. The directions of SNP effects on LTL and ALS were harmonized. The brief procedures are shown in the flowchart (Additional file 1: Fig. S1).

### Two-sample MR

The MR framework was based on the following three assumptions: *(1)* that the selected genetic variations are significantly associated with exposure; *(2)* that the selected genetic variations are not associated with other confounders; (*3)* that the selected genetic variations are significantly associated with the risk of outcome only in the pathway of exposure [19].

MR is a widely used genetic epidemiological approach, which assesses the causal relationship between exposures and outcomes by proposing naturally grouped risk alleles to simulate random allocation in RCTs. The multiplicative random effects IVW method was implemented as the main approach to examine the overall causal relationship between exposure and ALS based on the effects of IVs on LTL and ALS [20]. To validate the results from the IVW method, various additional MR methods were applied as sensitivity analyses, including the weighted median method, simple median method [21], MR Egger method [22] and MR-PRESSO method. The weighted median method gives consistent estimates when 50% of IVs are valid [21]. The MR Egger method and MR-PRESSO analysis were employed to test potential pleiotropy. The MR Egger method evaluates causal effects by adjusting for horizontal pleiotropy. The nonsignificant distance between its estimated intercept and the origin indicates the absence of pleiotropy. MR-PRESSO analysis was used to detect the influence of outliers [23]. The heterogeneity of IVs used in IVW estimates was tested by Cochran's Q test. Results with a P value smaller than 0.05 indicated the presence of heterogeneity. Leave-one-out analysis and single-SNP analysis were employed to evaluate the robustness of the significant results and the possibility of results being driven by a single SNP. We utilized the MR Steiger method to explore the potential reverse causal impact of ALS on exposure [24]. A publicly available online tool was adopted to calculate the statistical power of our analysis (https://shiny.cnsgenomics.com/mRnd/) [25]. All analyses were conducted using the “TwoSampleMR” package (version 0.5.6) [26] and the “MR-PRESSO” package (version 1.0) [23] in R 3.6.3 software. A P value less than 0.025 (0.05/2) after Bonferroni correction was considered to indicate a valid positive result. This study employed GWASs summary statistics, and an ethical permit is not required.

## Results

**Fig. 1:**
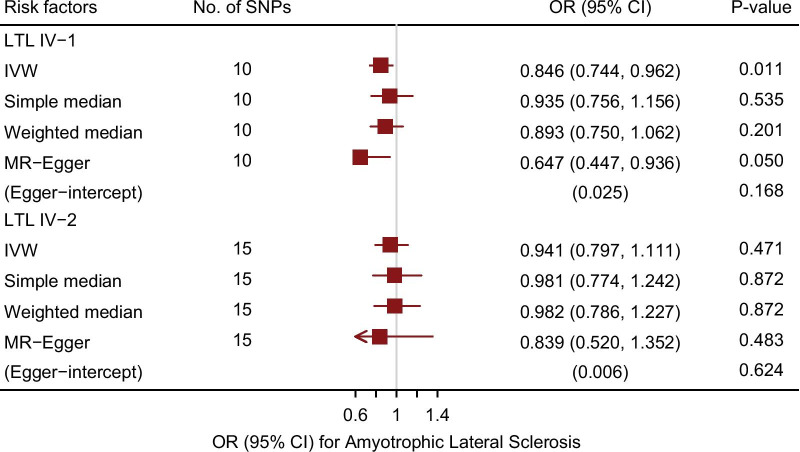
Association between genetically predicted leukocyte telomere length (LTL) and amyotrophic lateral sclerosis (ALS). Estimates are provided per approximately 1-standard deviation increase in LTL (bp). OR, odds ratio; CI, confidence interval; IVW, inverse variance weighted

IV-1 and IV-2 were included as proxies for LTL to investigate the relationship between LTL and ALS using various MR methods. The detailed results are shown in Table 1, and the main results are visualized in Fig. 1.

**Table 1:** Summary of the causal effects of each trait on ALS via different MR methods

		IV-1	IV-2
N SNPs		10	15
F statistics		954.49	1278.92
Simple median	OR (95% CI)	0.935 (0.756, 1.156)	0.981 (0.774, 1.242)
	*p* value	0.535	0.872
Weighted median	OR (95% CI)	0.893 (0.750, 1.062)	0.982 (0.786, 1.227)
	*p* value	0.201	0.872
MR Egger	OR (95% CI)	0.647 (0.447, 0.936)	0.839 (0.520, 1.352)
	*p* value	0.050	0.483
Inverse variance weighted	OR (95% CI)	0.846 (0.744, 0.962)	0.941 (0.797, 1.111)
	P value	0.011	0.471
MR Egger	intercept	0.025	0.006
	*p* value	0.168	0.624
Cochran’s Q	Q	5.176278	12.014047
	p value	0.82	0.61
MR-PRESSO	RSSobs	7.307	13.578
	*p* value	0.774	0.632
	outlier-corrected	NA	NA
MR Steiger	*p* value	0.0002	0.023
Statistical power		0.6	0.2

**Fig. 2:**
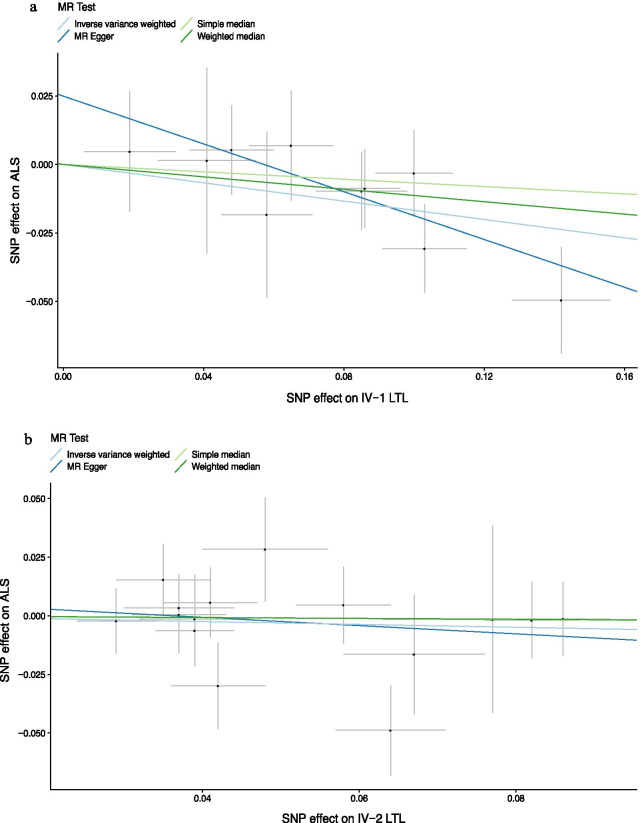
Scatter plot of single-nucleotide polymorphism (SNP) effects on leukocyte telomere length versus amyotrophic lateral sclerosis (ALS), with the slope of each line corresponding to the estimated Mendelian randomization (MR) effect per method. **a** Results based on IV-1; **b** Results based on IV-2

When we performed the two-sample MR analysis based on IV-1, a longer leukocyte telomere was inversely associated with the risk of ALS. The results of IVW analysis showed that the risk of ALS decreased by 15.4% (odds ratio (OR) = 0.846, 95% confidence interval (CI): 0.744–0.962, *P* = 0.011) with a genetically predicted one-standard deviation (1-SD) increase in LTL. This causal association was confirmed by the MR Egger method (OR = 0.647, 95% CI = 0.447–0.936, *P* = 0.050). Estimates based on the weighted median method (OR = 0.893, 95% CI: 0.750–1.062, *P* = 0.201) and simple median method (OR = 0.935, 95% CI: 0.756–1.156, *P* = 0.535) showed similar trends, despite statistical insignificance. The MR Egger intercept revealed no evidence of directional pleiotropy (intercept = 0.025, *P* = 0.168). There was no influence from outliers according to MR-PRESSO analysis. Cochran’s Q test indicated no heterogeneity. The MR Steiger test indicated that the causal estimates based on IV-1 for LTL affecting ALS followed the correct direction (*P* < 0.001). Scatter plots indicated the estimated effect of LTL on ALS by each SNP (Fig. 2a). Through single-SNP analysis, we found that this positive effect was mainly driven by rs9420907-C (OR = 0.706, *P* = 0.013) (Additional file 1: Fig. S2a). The results were further validated by leave-one out analysis (Additional file 1: Fig. S2b).

The results of IVW analysis indicated that the OR of LTL IV-2 for ALS was 0.941 (95% CI: 0.797–1.111, P = 0.471), suggesting a possible decrease in the risk of ALS by 5.9% due to a 1-SD increase in telomere length. The result is similar to those obtained in the sensitivity analyses (Table 1). No obvious horizontal pleiotropy interference was indicated by the MR Egger intercept (intercept = 0.006, *P* = 0.624). Neither the influence of outliers nor heterogeneity was found. There was little evidence that increased LTL was associated with a decreased risk of ALS based on IV-2. However, a significant impact of rs9419958-T on ALS was highly suggested in the single-SNP analysis (OR = 0.466, *P* = 0.015) (Additional file 1: Fig. S3).

Furthermore, we estimated the linkage disequilibrium (LD) of rs9420907 in IV-1 and rs9419958 in IV-2 using the publicly available online tool SNIPA (http://snipa.helmholtz-muenchen.de/snipa3/index.php). We found that both were localized at the OBFC1 locus with r^2^ equal to 1, indicating that they are strongly genetically linked.

## Discussion

In the present study, we found that a longer leukocyte telomere may be a protective factor for ALS in the European population using a two-sample MR approach. Given the close relationship between LTL and ageing, the results support the long-held view that ageing is associated with ALS [27]. Our findings suggest that LTL may contribute to the complex pathogenesis of ALS. LTL could be a potential marker for the prediction of ALS. Decelerating LTL loss may be a new strategy for the prevention of ALS.

We evaluated the causal relationship between LTL and ALS. In MR analysis of IV-1, we found that the risk of ALS was reduced by 15.4% (OR = 0.846, 95% CI: 0.744–0.962, *P* = 0.011) for every genetically predicted 1-SD increase in LTL. It was initially reported that 1 SD represented approximately 650 base pairs in LTL [11], which is nearly equivalent to the loss of LTL from 26 years of natural ageing in the European population [28]. In our analysis of IV-2, this trend seemed weak (OR = 0.941, 95% CI: 0.797–1.111, *P* = 0.471). Nevertheless, the results generated from IV-1 were regarded as more reliable for the following reasons. First, the GWAS enrolled for IV-1 was of higher quality than IV-2. The method adopted for measuring LTL was Southern blotting, which is the gold standard for LTL measurement [13]. However, the method used for measuring LTL in IV-2 was quantitative PCR, which shows large variations among different studies and only provides the average telomere length as a relative ratio [29]. Similarly, the strict Bonferroni method was adopted in IV-1 GWAS for P-value correction. In LTL IV-2 GWAS, the FDR method, which is less strict, was used for P value correction instead of the strict Bonferroni method. Second, the proportion of phenotypic variance explained by IV-1 was higher than that explained by IV-2; thus, the statistical power of analysis with IV-1 was 40% higher than that of analysis with IV-2 (Table 1). Recently, the study published by Gao et al. reported that LTL had no direct causal effect on ALS and suggested that shorter LTL can indirectly reduce ALS risk [30]. Their study was based on IV-2 and an ALS GWAS involving 37,684 individuals of European ancestry [31]. Due to the less strict LTL measurement and P value correction method of IV-2 GWAS, low proportion of LTL phenotypic variance explanation of IV-2, and the relatively limited number of participants in the ALS GWAS they utilized, the statistical power of that study was declared to be less than 30%. Altogether, the causal effect of IV-1 on ALS is more reliable owing to its comprehensive data source. We performed internal and external verification to replicate the causal link between LTL and ALS generated by IV-1. Various sensitivity analyses were utilized as internal validation to ensure the robustness of the causal link between IV-1 and ALS. This causal association was confirmed by the MR Egger method (OR = 0.647, 95% CI = 0.447–0.936, *P* = 0.050), which is an approach to evaluate causal effects by adjusting for horizontal pleiotropy at the cost of statistical power. Similarly, external validation was conducted with the second-largest European-based ALS GWAS summary-level data (cases = 12,577; controls = 23,475) [31]. We found that increased LTL was a protective factor against ALS (OR-IVW = 0.974, 95% CI = 0.951–0.997, *P* = 0.028). However, the statistical power is as low as 10%. All the statistics were double-checked. In addition to the difference in sample size, there is another reason proposed for the lack of true replication. A total of 10,031,630 genotyped and imputed variants were available in the ALS GWAS we utilized in our study previously for the downstream association analysis [17]. The ALS GWAS for replication provided 869, 7640 genetic variants, which estimated a lower SNP-based heritability [31]. Therefore, further replication is still needed when GWAS with more participants and genotyped variants is available.

The exact underlying mechanism linking LTL to ALS is still unclear. Both leave-one-out analysis and single-SNP analysis of IV-1 and IV-2 indicated that the OBFC1 (oligonucleotide/oligosaccharide-binding fold containing one) locus had a strong effect on ALS. The OBFC1 protein is part of the TPP1 protein complex that interacts with telomerase and telomere ssDNA-binding proteins. It participates in maintaining telomere integrity and downregulating telomerase action [32–34]. Overexpression of truncated mutants in OBFC1 leads to telomere elongation in cancer cells [32]. However, no study has explored the effect of OBFC1 in ALS. Because longer telomeres appeared to be a promising marker for the prognosis of ALS, extending the median survival time by 16% [7], we hypothesized that this genotype may be a protective indicator for ALS. Similarly, it may be a predictor of a slow pattern of ALS progression in that its function could be related to the onset and development of ALS. More clinical studies and animal experiments are needed to verify this hypothesis. A future next step would be to investigate the relationship between OBFC1 and ALS incidence as well as prognosis and the function of OBFC1 in disease-model mice and cells.

Furthermore, LTL is a solid marker of ageing [5]. Ageing is also thought to share common pathologic pathways with ALS, which may provide insights into the causality between LTL and ALS. Compared with those of motor neurons from age-matched healthy controls, the transcriptomes of motor neurons differentiated from pluripotent stem cells of ALS patients are more similar to those of older neurons [35]. Direct evidence has suggested that the common C9orf72 hexanucleotide repeat expansion in ALS can form a stable G-quadruplex involved in the regulation of telomere integrity and ageing [36]. Many ALS disease-causing genes, including OPTN, TBK1 and SOD1, play important roles through the autophagy/lysosomal degradation pathway, which is of great significance in ageing [37]. Cell proliferative activity and the ability to cope with oxidative stress, excitatory cytotoxicity, and apoptosis play vital roles in ALS and ageing [38].

In addition, other mechanisms may participate in the protective effect of LTL on ALS. Population-based research demonstrated that telomere length displayed sex differences [39], partly because oestrogen directly activates a promoter of telomerase [40] and enhances the activation of telomerase through the phosphoinositol-3-kinase/Akt [41] and nitric oxide pathways [42], leading to decelerated telomere shortening. According to our results, a longer telomere will decrease the risk of ALS, which is consistent with the higher prevalence of ALS in males is than in females [43]. We deduced that oestrogen may further assist in the role of telomeres in ALS. Oestrogen supplementation may have a positive effect on ALS. Animal experiments proved that extra 17β-oestradiol (known as the most potent form of oestrogen) had a promising influence on ALS, improving motor performance in male SOD1 G93A mice [44] and delaying disease progression in ovariectomized mice [45]. Although oestrogen replacement treatment is associated with attenuated motor symptoms in Parkinson’s disease [46], high-quality clinical trials on ALS need to be conducted. Similar to medicated interventions that slow telomere shortening, some effective methods to delay telomere shortening, such as lowering stress and consuming a high-quality diet (e.g., intaking ω-3 free fatty acids and some antioxidants, with low consumption of saturated fat) [47], are also worth trying in the further exploration of ALS treatments. Nonetheless, given the high metabolism of ALS patients, the impact of low intake of saturated fat is still unknown. Altogether, these findings may provide new ideas for disease management in the future.

The results of our study are reliable since we enrolled the largest current GWASs to explore the causal relationship between LTL and ALS. In addition, the MR framework utilized in our study minimized interference from confounders and reverse causality. Moreover, our study was conducted in a unitary race, and the heritability of exposure was impressive. However, there were still some limitations in our study: (1) although both IV-1 and IV-2 confirmed the important role of the OBFC1 gene in ALS, the results produced by IV-1 were not completely replicated by IV-2. Because of the comprehensive data source, results generated with IV-1 are more reliable and recognized as the main results of our study. To replicate this thoroughly, larger GWASs are needed in the future; (2) the U-shaped relationship could not be estimated because of the principle of MR that the risk of disease is linearly related to telomere length, and (3) although the relationships between sex, LTL, and ALS have been discussed, we could not investigate the sex-specific effects of LTL on ALS because of the absence of available corresponding GWASs.

## Conclusion

Our study suggests that an increased LTL has a causal relationship with ALS in the European population, mainly based on an LTL-related GWAS with 9190 individuals, and underscores the importance of protecting against telomere loss in ALS.

## Supplementary Information

**Additional file 1. Fig. S1**. Flow chart for dataset preparation for MR analyses.** Fig. S2**. The effect of single nucleotide polymorphisms (SNPs) on amyotrophic lateral sclerosis (ALS) based on IV-1.** Fig. S3**. Association between leukocyte telomere length (LTL) and amyotrophic lateral sclerosis (ALS) measured by single nucleotide polymorphisms (SNPs) based on IV-2.** Table S1**. Characteristics of instrumental variables (IVs) associated with leukocyte telomere length.

## Data Availability

All data generated or analyzed during this study are included in this published article and its supplementary information files. Codes generated or used during the study are available from the corresponding author by request.

## Abbreviations

ALS: Amyotrophic lateral sclerosis
LTL: Leukocyte telomere length
MR: Mendelian randomization
GWAS: Genome-wide association study
IVW: Inverse variance weighted method
IV: Instrumental variable
SD: Standard deviation
LD: Linkage disequilibrium
CI: Confidence interval
BMI: Body mass index
SNP: Single-nucleotide polymorphism
OR: Odds ratio
OBFC1: Oligonucleotide/oligosaccharide-binding fold containing one
