# Supplementary material for: Leukocyte telomere length and amyotrophic lateral sclerosis: a Mendelian randomization study
Source: Orphanet J Rare Dis. 2021 Dec 14;16:508. doi: 10.1186/s13023-021-02135-2 (PMC8670150; doi:10.1186/s13023-021-02135-2)
Supplement: Supplementary file 1 — Additional file 1. Fig. S1. Flow chart for dataset preparation for MR analyses. Fig. S2. The effect of single nucleotide polymorphisms (SNPs) on amyotrophic lateral sclerosis (ALS) based on IV-1. Fig. S3. Association between leukocyte telomere length (LTL) and amyotrophic lateral sclerosis (ALS) measured by single nucleotide polymorphisms (SNPs) based on IV-2. Table S1. Characteristics of instrumental variables (IVs) associated with leukocyte telomere length. [file 13023_2021_2135_MOESM1_ESM.docx]

**Additional File**

**Leukocyte telomere length and amyotrophic lateral sclerosis: a Mendelian randomization study**

Kailin Xia^a,b^, Linjing Zhang^a,b^, Gan Zhang^a,b^, Yajun Wang^a,b^, Tao Huang*^,c,d^, Dongsheng Fan*^,a,b,e^

^a^Department of Neurology, Peking University Third Hospital, Beijing, China

^b^Beijing Municipal Key Laboratory of Biomarker and Translational Research in Neurodegenerative Diseases, Beijing, China

^c^Department of Epidemiology and Biostatistics, School of Public Health, Peking University, Beijing, China

^d^Key Laboratory of Molecular Cardiovascular Sciences (Peking University), Ministry of Education, Beijing, China

^e^Key Laboratory for Neuroscience, National Health Commission/Ministry of Education, Peking University, Beijing, China

*These authors contributed to this work equally

**Corresponding authors**

Tao Huang, huangtao@bjmu.edu.cn

Dongsheng Fan, dsfan@sina.com

Postal address: 49 North Garden Road, Haidian District, 100191, Beijing, China

**Supplementary Fig. 1** Flow chart for dataset preparation for MR analyses

GWAS, genome-wide association study; MR, Mendelian randomization; ALS, amyotrophic lateral sclerosis


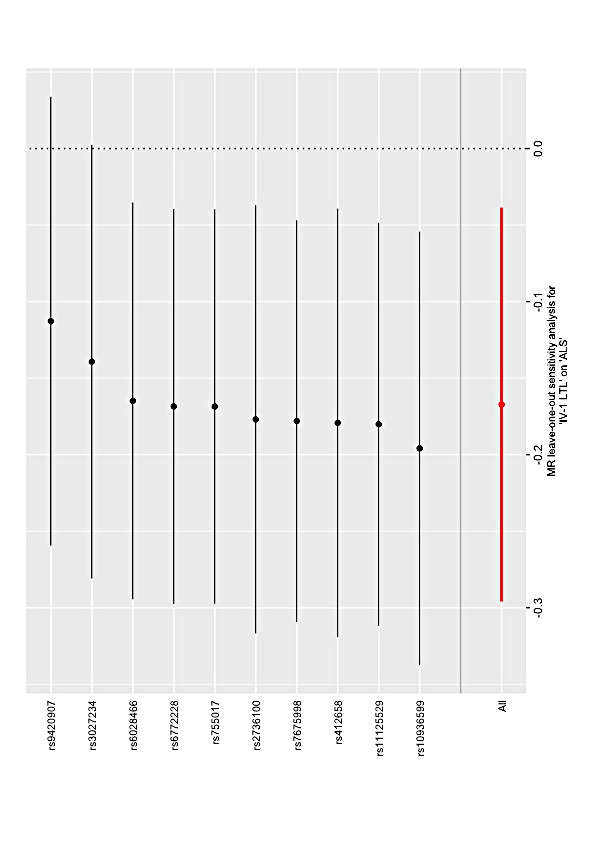

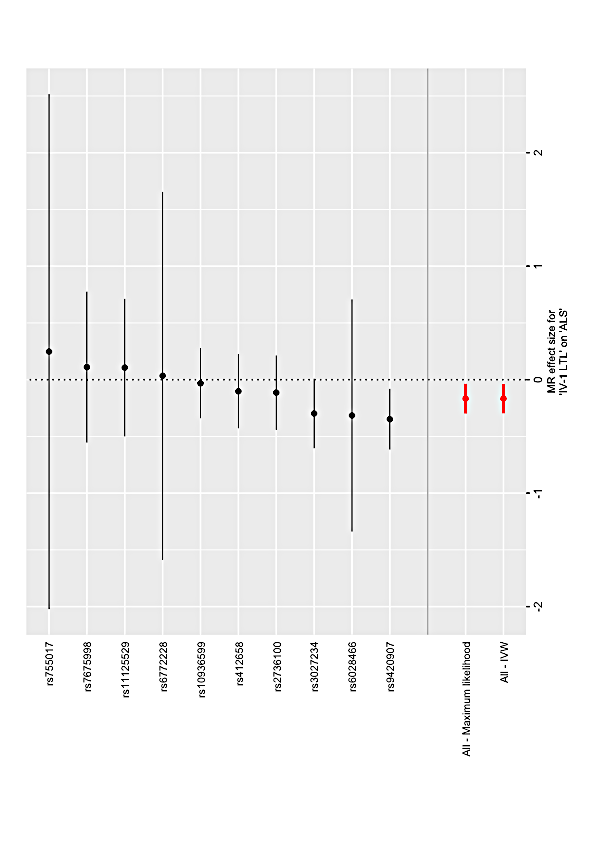


b

a

**Supplementary Fig. 2** the effect of single nucleotide polymorphisms (SNPs) on amyotrophic lateral sclerosis (ALS) based on IV-1

Round dots represent the odds ratio of ALS per genetically predicted 1 standard deviation (SD) increase in LTL; horizontal lines represent 95% confidence intervals (CIs).

a Association between leukocyte telomere length (LTL) and ALS measured by single SNPs

b Leave-one-out analysis of leukocyte telomere length (LTL) in ALS


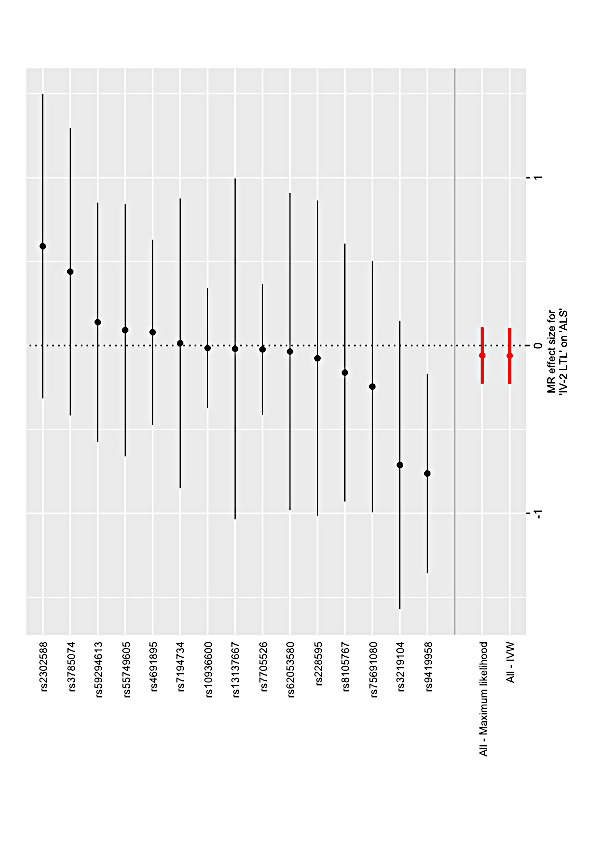


**Supplementary Fig. 3** Association between leukocyte telomere length (LTL) and amyotrophic lateral sclerosis (ALS) measured by single nucleotide polymorphisms (SNPs) based on IV-2

Round dots represent the odds ratio of ALS per genetically predicted 1 standard deviation (SD) increase in LTL; horizontal lines represent 95% confidence intervals (CIs)

**Supplementary Table 1.** Characteristics of instrumental variables (IVs) associated with leukocyte telomere length.

|  | | | | | | | |
| --- | --- | --- | --- | --- | --- | --- | --- |
| SNP | a1 | a2 | a1_freq | beta | SE | p-value | Traits |
| rs11125529 | A | C | 0.16 | 0.065 | 0.012 | 6.06E-03 | IV-1 LTL |
| rs6772228 | T | A | 0.87 | 0.041 | 0.014 | 4.97E-02 | IV-1 LTL |
| rs12696304 | C | G | 0.74 | 0.09 | 0.011 | 5.41E-08 | IV-1 LTL |
| rs10936599 | C | T | 0.76 | 0.1 | 0.011 | 1.76E-09 | IV-1 LTL |
| rs1317082 | A | G | 0.71 | 0.097 | 0.011 | 4.57E-09 | IV-1 LTL |
| rs10936601 | C | T | 0.74 | 0.087 | 0.011 | 8.64E-08 | IV-1 LTL |
| rs7675998 | G | A | 0.8 | 0.048 | 0.012 | 1.00E-02 | IV-1 LTL |
| rs2736100 | C | A | 0.52 | 0.085 | 0.013 | 2.14E-05 | IV-1 LTL |
| rs9419958 | T | C | 0.13 | 0.129 | 0.013 | 5.26E-11 | IV-1 LTL |
| rs9420907 | C | A | 0.14 | 0.142 | 0.014 | 1.14E-11 | IV-1 LTL |
| rs4387287 | A | C | 0.14 | 0.12 | 0.013 | 1.40E-09 | IV-1 LTL |
| rs3027234 | C | T | 0.83 | 0.103 | 0.012 | 2.75E-08 | IV-1 LTL |
| rs8105767 | G | A | 0.25 | 0.064 | 0.011 | 1.00E-03 | IV-1 LTL |
| rs412658 | T | C | 0.35 | 0.086 | 0.01 | 1.83E-08 | IV-1 LTL |
| rs6028466 | A | G | 0.17 | 0.058 | 0.013 | 4.00E-03 | IV-1 LTL |
| rs755017 | G | A | 0.17 | 0.019 | 0.013 | 3.40E-01 | IV-1 LTL |
| rs3219104 | C | A | 0.83 | 0.042 | 0.006 | 9.60E-11 | IV-2 LTL |
| rs10936600 | T | A | 0.24 | -0.086 | 0.006 | 7.18E-51 | IV-2 LTL |
| rs4691895 | C | G | 0.78 | 0.058 | 0.006 | 1.58E-21 | IV-2 LTL |
| rs7705526 | A | C | 0.33 | 0.082 | 0.006 | 5.34E-45 | IV-2 LTL |
| rs59294613 | A | C | 0.29 | -0.041 | 0.006 | 1.17E-13 | IV-2 LTL |
| rs9419958 | C | T | 0.86 | -0.064 | 0.007 | 5.05E-19 | IV-2 LTL |
| rs228595 | A | G | 0.42 | -0.029 | 0.005 | 1.43E-08 | IV-2 LTL |
| rs2302588 | C | G | 0.1 | 0.048 | 0.008 | 1.68E-08 | IV-2 LTL |
| rs7194734 | T | C | 0.78 | -0.037 | 0.006 | 6.94E-10 | IV-2 LTL |
| rs8105767 | G | A | 0.3 | 0.039 | 0.005 | 5.42E-13 | IV-2 LTL |
| rs75691080 | T | C | 0.09 | -0.067 | 0.009 | 5.99E-14 | IV-2 LTL |
| rs55749605 | A | C | 0.58 | -0.037 | 0.007 | 2.45E-08 | IV-2 LTL |
| rs13137667 | C | T | 0.96 | 0.077 | 0.014 | 2.43E-08 | IV-2 LTL |
| rs34991172 | G | T | 0.07 | -0.061 | 0.011 | 6.19E-09 | IV-2 LTL |
| rs2736176 | C | G | 0.31 | 0.035 | 0.006 | 3.53E-10 | IV-2 LTL |
| rs3785074 | G | A | 0.26 | 0.035 | 0.006 | 4.64E-10 | IV-2 LTL |
| rs62053580 | G | A | 0.17 | -0.039 | 0.007 | 4.08E-08 | IV-2 LTL |
| rs34978822 | G | C | 0.02 | -0.14 | 0.023 | 7.26E-10 | IV-2 LTL |
| rs73624724 | C | T | 0.13 | 0.051 | 0.007 | 6.33E-12 | IV-2 LTL |
| rs2853677 | A | G | 0.59 | -0.064 | 0.006 | 3.35E-31 | IV-2 LTL |

Abbreviation: a1: effect allele; a2; the other allele; a1_freq: the frequency of a1; SE: standard error
